# Meeting Student Mental Health Needs in Survival Mode: A Qualitative Analysis of School Professional Mental Health Delivery at Michigan High Schools in Response to COVID-19

**DOI:** 10.1007/s12310-025-09838-y

**Published:** 2026-01-13

**Authors:** Amy Rusch, Sarah M. Stilwell, Alex Ammann, Seo Youn Choi, Shawna N. Smith

**Affiliations:** 1 Department of Health Behavior and Health Equity, University of Michigan School of Public Health, Ann Arbor, MI USA; 2 Department of Health Management and Policy, University of Michigan School of Public Health, Ann Arbor, MI USA; 3 Department of Psychiatry, University of Michigan Medical School, Ann Arbor, MI USA

**Keywords:** Mental health, COVID-19, Evidence-based practice, CBT, School-based services, Qualitative

## Abstract

The COVID-19 pandemic significantly disrupted many school-based services, including mental health support. This qualitative study examines the efforts of school professionals from sixteen Michigan high schools participating in a larger trial implementing a mental health evidence-based practice program to continue delivery in response to COVID-19 related school building closures. Nineteen in-depth interviews were carried out between June and October 2021 to understand to what extent and through what mechanisms school professionals modified and continued their mental health service delivery. Findings from thematic analysis reveal heightened responses to changes in student mental health needs through multiple modalities, modification of previous mental health service delivery methods to fit virtual and hybrid context, and shifts in school professional roles while simultaneously managing providers’ personal responses to COVID-19. Themes identified underscore the complexity of sustaining mental health service provision amid school building closures and virtual or hybrid learning environments. While findings highlight challenges related to student accessibility, engagement, and communication, the study also identified unanticipated positive shifts in delivery, including increased service utilization by students who previously did not access school-provided mental health services and, in some schools, increased collaboration with teachers and administrators. Findings illuminate how schools and individuals delivering mental health services respond to crises and can inform the development of strategies to improve the resilience and adaptability of school-based mental health services.

**Supplementary Information:**

The online version contains supplementary material available at 10.1007/s12310-025-09838-y.

Tasked with meeting the significant and rising mental health needs of adolescents, schools have become increasingly important providers of mental health services (Hoover & Bostic, 2021; Weist et al., 2023). Depression and anxiety disorders affect approximately 15% and 30% of school-aged youth respectively, and those numbers are expected to increase (Lebrun-Harris et al., 2022; Merikangas et al., 2010). As their need for mental health care continues to rise, ensuring access to mental health treatment is crucial for supporting youth who would benefit from mental health evidence-based practices (EBPs) (García-Carrión et al., 2019). In the United States, approximately 60% of K-12 students who receive any mental health services report receiving some services in school, and nearly 40% of these students reported receiving school services exclusively (National Center for Education Statistics, 2024). Schools are often the first, if not only, point of connection to mental health care, and thus critically serve the growing number of students in need (Ali et al., 2019; Kern et al., 2017; Stempel et al., 2019).

## COVID-19 and Student Mental Health

The COVID-19 pandemic and its resultant school building closures posed unprecedented challenges for school-based mental health care delivery (Chaabane et al., 2021; Pfefferbaum, 2021). Standard mechanisms and protocols for connecting students to school-provided mental health support were disrupted; students lost access to services many had relied on (Naff et al., 2020, 2022; Richards et al., 2023). Concurrently, students experienced significant mental health challenges during the pandemic and reported feeling more anxious, depressed, fatigued, and distressed (Elharake et al., 2022; Schwartz et al., 2021). The challenges to school mental health delivery disrupted school-based interventions when many adolescents needed those services more than ever (Naff et al., 2022).

In the years following the COVID-19 pandemic, research efforts investigated the extent to which COVID-19 impacted student mental health needs (Hawrilenko et al., 2021; Pfefferbaum, 2021; Viner et al., 2022). Beyond underscoring pandemic-related effects on student mental health, researchers sought to understand concurrent and subsequent modifications to mental health care made across healthcare settings (Raphael et al., 2021). This included understanding changes in support for specific aspects of the school-based mental health delivery implementation process, for example through training and technical assistance (Olson et al., 2021). Researchers have examined how school-based therapists and psychologists changed practices to support students during the onset of COVID-19 related school closures, within a state, across states, and across countries (Marraccini et al., 2023; Reupert et al., 2022; Robinson-Williams, 2024). Other studies have explored the impact of COVID-19 on school mental health and implementation from a policy and practice perspective (Hoagwood et al., 2020; Palinkas et al., 2021; Short et al., 2022).

Unlike previous studies that have primarily analyzed broader policy implications or general shifts in practice, our study delves into the why and how behind the adjustments to mental health delivery within the context of a specific mental health EBP. By investigating both mental health delivery changes in relation to one EBP and the underlying mechanisms driving mental health delivery changes more broadly, we seek to offer more descriptive insights into the process of modifying mental health EPB delivery, and mental health delivery overall, in response to real-time disruptions.

Understanding how mental health service delivery in schools changed following the COVID-19 pandemic is relevant for educators, researchers, implementers, and policymakers. Insight into modifications made during this period allows these groups to more effectively prepare for potential disruptions that could necessitate similar changes or future adjustments to modes of delivery (Naff et al., 2020). School officials and mental health providers were forced to quickly make decisions that varied from established practices with little guidance or support (McLeod & Dulsky, 2021). These actions illuminate the adaptability and resilience of the school mental health care system in times of crisis (Song et al., 2020). While this research was conducted in reaction to a unique event, it offers a crucial example of the flexibility mental health delivery requires to respond to unexpected circumstances and meet the evolving needs of rapidly changing approaches to mental health care in schools.

## Context of the Current Study

The current study was administered within the context of a cluster-randomized implementation trial. The Adaptive School-based Implementation of CBT (ASIC) study sought to evaluate school-level adaptive implementation strategies to support Cognitive Behavioral Therapy (CBT) delivery in high schools across Michigan. This study was in its second year of data collection, and only a few weeks from study completion, when COVID-19 disrupted study goals (Kilbourne et al., 2018). Prior to COVID-19-related school building closures, school professionals (SPs) had been trained and received support for CBT delivery to their students and 91% of SPs reported CBT delivery at least once during the study (Smith et al., 2022). However, following school building closures, the extent to which SPs participating in ASIC could and would continue delivering CBT to their students remained in question. The current research focused on ASIC-enrolled SPs who were successfully delivering CBT to students prior to COVID-19 school building closures to understand if and how SPs sustained or modified their CBT delivery to students.

While this research was not designed within the ASIC study originally, it inadvertently became a quasi-natural experiment due to the COVID-19 pandemic. This unforeseen disruption to planned study activities offered a unique opportunity to document and analyze subsequent modifications. SPs had to make rapid decisions regarding their roles in supporting student mental health without pre-existing frameworks or guidance; to continue CBT delivery required significant improvisation and creativity. Accordingly, this scenario provides a valuable case study and captures an unprecedented time that necessitated dynamic changes in mental health service delivery. By working with a group of SPs contemporaneously engaged in the ASIC study’s CBT training and delivery, this research offers in-depth insights into how these professionals approached challenges posed by the rapid transition from in-person to remote mental health interventions. Due to the context in which the ASIC study occurred and the ability to engage with a group of SPs who had been engaged in in-person mental health EBP delivery, this study is uniquely positioned to complement the discourse about modifying school mental health delivery from a provider perspective.

### Specific Aims

Given the increased mental health needs for students during the COVID-19 pandemic, this study seeks to address this gap in knowledge by utilizing SP perspectives to examine how schools adapted to the changing circumstances to provide mental health services to their students during the COVID-19-related disruptions. Specifically, this study aims to answer these research questions and sub-aims:

***Research Question 1***. To what extent were SPs able to continue delivering CBT and other mental health services to students, both formally and informally, in response to COVID-19 related school building closures?

**Sub-aim (A)** Identify the factors that contributed to the ability or inability of SPs to continue delivering CBT and other mental health services to students during COVID-19-related school closures.

**Sub-aim (B)** Examine what modifications or specific changes enabled SPs who successfully continued delivering mental health support to effectively address student needs despite the challenges posed by COVID-19.

***Research Question 2***. What barriers and facilitators impacted the ability of SPs to support the continued delivery of CBT or other mental health support in the wake of COVID-19?

These questions are key for understanding SP experiences that can inform schools and help establish plans for mental health service adaptation in future instances requiring remote delivery. Ultimately, this research seeks to leverage this knowledge to better understand how schools and SPs respond to exogenous shocks impacting the usual delivery of mental health support. These findings have implications for the future development of strategies, best practices, and policies regarding school-provided mental health services delivery during future public health crises or other changes to delivery contexts.

## Methods

### The ASIC Study

Data were collected as part of the ASIC study, an NIH type III hybrid implementation effectiveness trial (Kilbourne et al., 2018). The overall goal of the ASIC study was to compare the effectiveness of different sequences of implementation strategies for SP’s delivery of CBT in the school setting. The implementation support, including SP training and materials, were developed and delivered by Transforming Research into Action to Improve the Lives of Students (TRAILS). TRAILS strives to improve youth access to evidence-based mental health services by training school mental health professionals in effective practices (e.g., CBT) (Meyer et al., 2024; TRAILS to Wellness, 2025). CBT is an evidence-based psychotherapy which has been validated to improve mental health outcomes for a variety of disorders, including anxiety and depression for youth (Arnberg & Öst, 2014) and considered appropriate for mild cases of depression and anxiety (Flynn & Warren, 2014; Gautam et al., 2020; Koschmann et al., 2019). SPs were encouraged to deliver CBT components including psychoeducation, relaxation, mindfulness, cognitive restructuring, behavioral activation, and exposure (Koschmann et al., 2019). The ASIC study was approved by the University of Michigan Institutional Review Board. Further information on the ASIC study can be found elsewhere (Kilbourne et al., 2018; Smith et al., 2022).

Ninety-four high schools in Michigan participated in the ASIC study and 169 study-enrolled SPs at these schools were trained and received implementation support for CBT delivery between January 2019 and December 2019 (Phases 1 and 2). SPs received no active support from January 2020 (Phase 3, or Sustainment Phase) and were expected to continue CBT delivery and report their weekly delivery until April 2020. On March 16, 2020, however, the COVID-19 pandemic led to statewide in-person school building closures across Michigan resulting in unprecedented changes to in-person provision of student mental health support, including CBT by SPs. From March 2020, if SPs were delivering any CBT at all, they had to modify their delivery due to school building closures and use of virtual learning environments.

In response to COVID-19 and related school closures, TRAILS (outside of the ASIC study) administered a web-based needs assessment survey in April 2020 with school mental health providers who previously participated in the TRAILS program (Rusch et al., 2021). Based on these responses, TRAILS quickly developed a skills-based group program, Coping with COVID-19 (CC-19), which was guided by CBT and mindfulness principles (Rodriguez-Quintana et al., 2021). TRAILS provided shared resources to support SPs and students including access to online materials and 3-hour virtual training for the CC-19 program, training and self-care materials for SPs, instructional guide for delivering virtual groups, clinical consultation, and other materials related to COVID-19 (Rodriguez-Quintana et al., 2021). While TRAILS provided materials and recommendations that could be applied to virtual CBT delivery, the approaches SPs used to modify service delivery in practice were generally left to their professional judgment and capacity to enact recommended changes.

### Present Study

In response to COVID-19, the ASIC study team planned a series of qualitative interviews post-school building closures with a subsample of SPs participating in the ASIC study. The focus of these research questions was on exploring types of modifications to CBT delivery in relation to sustainment. As a result, only SPs who already reported successfully delivering some form of CBT before building closures and potentially had the ability to modify their approach comprised this sample. While not originally part of the ASIC study protocol, these semi-structured, in-depth interviews were added post hoc to capture the changes to the intervention and SP mental health delivery in a new and often difficult context. The interview guide (Supplemental Table 1) included questions to understand: sustained use of TRAILS materials and/or CBT delivery during COVID-19, if any; barriers and/or facilitators to sustained use of CBT by SPs during COVID-19 related school building closures; mechanisms that allowed SPs to transition mental health EBP delivery to a virtual learning environment; and effects of virtual learning environments on CBT delivery. Approval for this amendment to the study protocol was granted by the University of Michigan Institutional Review Board.

SPs enrolled in the ASIC study who adopted and were delivering CBT to students prior to COVID-19 school building closures, defined as those who delivered CBT for 6 or more weeks during the Sustainment Phase (Phase 3; 10 weeks) of the ASIC study, were invited to participate in interviews. The 58 ASIC-enrolled SPs that had adopted CBT before the onset of the pandemic were invited via email to complete interviews with one of two members of the study team. Phone follow-up using office phone numbers collected as part of the ASIC study consent process was not possible due to many SPs working from home at the point of contact. SPs were contacted every two weeks until they accepted, declined, or had been contacted a total of four times.

Interviews with participating SPs were carried out between June and October 2021 and were conducted by two study team members familiar with the ASIC study and with training in conducting semi-structured interviews. Interviews were conducted via the Zoom video conferencing platform and, as they were virtual, SPs could attend from any location; however, the study team requested that interviews be conducted in a private location with reliable internet access. Before beginning, SPs provided verbal consent to participate and be recorded. SPs were asked to reflect on three different time periods in their interviews: immediately following COVID-19 related school shutdowns through the end of the 2019/2020 academic year, the 2020/2021 academic year when a majority of schools were offering some form of hybrid educational delivery or a mix of in person and virtual academic instruction, and the preparation for and early weeks of the 2021/2022 academic year when all schools were returning to in-person instruction. SPs were provided with a $25 incentive for participation. Following interview completion, recordings were downloaded from Zoom, de-identified, transcribed by Rev.com, and then deleted from the Zoom platform. The transcripts were then confirmed by the study team to ensure accuracy of the transcription service.

### Analysis

The methodological design of this study sought to elucidate the process of adapting mental health service delivery, specifically CBT, in the school setting in response to an exogenous shock (e.g., COVID-19, other public health crisis). The analysis was guided by thematic analysis, which provided a framework for identifying, organizing, and interpreting patterns and key themes across the data relevant to the research questions (Braun & Clarke, 2006). To enhance analytical depth and the classification of identified themes, the research team integrated grounded theory principles to refine the coding structure and ensure that emergent themes were grounded in participants’ accounts and derived from empirical data (Charmaz, 2005; Khan, 2014).

### Analysis Plan

Three main stages guided data analysis. First, the first author read through all interviews in their entirety as well as brief memos that were written by the interviewer immediately following interviews to capture primary interview themes, places where saturation had been reached, and points requiring additional probing in future interviews. The purpose of this step was to identify similar findings in the data for more in-depth analysis in future stages.

Second, an initial codelist was developed by the first author from these data sources using an inductive, data-driven approach characteristic of thematic analysis. The initial codelist was informed by the six C’s of grounded theory: causes, contexts, contingencies, consequences, co-variances, and conditions to structure code categorization (Corbin & Strauss, 2015). Substance codes were categorized and codes within each category were compared and defined to eliminate duplication. The initial codelist was then tested by the first author with three randomly chosen transcripts for accuracy and completeness. Points that were not captured by the initial codelist were identified and inductive codes encapsulating these contributions were added to the code list before being finalized through team discussion.

Third, codes were applied to all 19 interviews using open coding, an inductive approach to descriptively code interview transcripts and identify patterns and themes. All interviews were coded independently by two members of the research team. Coding discrepancies were discussed in regular meetings and resolved by consensus, with guidance from the senior author as needed. Once all interviews were coded, NVivo 12 qualitative software was used to identify and correct duplicate codes (QSR International, 2017). All final codes were examined by the first author and substance codes that had frequencies fewer than four were either deleted or merged. Using a bottom-up approach, substance codes were then grouped to create subthemes of related ideas. Subthemes were again reviewed by the first author and combined to create overarching themes. These themes and subthemes were refined using a collaborative, iterative process involving all members of the coding team until all authors approved of final themes, subthemes, and definitions. Rigor was supported through an audit trail and an analytical journal documenting the analytic decision-making process.

## Findings

### Sample Characteristics

#### School Professionals

Of the 58 SPs invited to participate in qualitative interviews, *N* = 19 SPs (33%) representing 16 schools ultimately completed an interview. Interviews lasted between 35 and 63 min and averaged 47 min. The sample was predominantly female (*N* = 17; 89%) and white (*N* = 18; 94%). Almost half of participants served in a rural school district and about half had no training in CBT prior to ASIC study participation. Participant characteristics and additional metrics can be found in Table 1.

**Table 1:** Study participant characteristics

SP-level (*N* = 19)	Percentage (*N*)/Mean (SD; Range)
Served in rural school district (vs. non-rural)	47% (9)
White or Caucasian (vs. non-white)	95% (18)
Female (vs. male)	90% (17)
Years in current position	8 years (SD = 7; 1–22)
Professional role	
School Counselor	58% (11)
School Social Worker	37% (7)
Behavioral Intervention Specialist	5% (1)
Prior CBT training	
Yes, formal (i.e., graduate program)	21% (4)
Yes, informal only	32% (6)
No	47% (9)
CBT training in graduate school	
No graduate training	0%
1–2 lectures on CBT as part of a larger course	21% (4)
Quarter/semester course exclusively on CBT	0%
Didactic course on CBT with associated clinical supervision of one or more cases	0%
Professional development on CBT in the past 5 years (Choose all that apply)	
None	58% (11)
Brief presentation or workshop	26% (5)
Self-directed (e.g., online course, reading, YouTube videos)	21% (4)
1-to-2-day training	16% (3)
Mentioned in other classes/trainings	5% (1)
Training with follow-up consultation or supervision of a case	0%

19 SPs comprised the final interview sample

#### Schools

The 19 SPs interviewed represented 16 of 94 ASIC-participating schools. Within the larger implementation trial, schools were categorized based on certain characteristics including student body population size, percentage of students eligible for free or reduced school lunch, and school’s geographic classification as rural or non-rural. SPs interviewed constituted a fairly representative selection of schools in terms of size (56% >500 students) and school location (56% rural), but only three schools (19%) were represented that had ≥ 50% students eligible for free or reduced lunch (Table 2).

**Table 2:** School-level characteristics of interviewed school professionals

School-level (*N* = 16)	Mean (SD; Range)/Percentage (*N*)
School size: Number of Students	810 students (SD = 610; 66 − 2,025)
Free/reduced lunch: % of students eligible	40% of students (SD = 15%; 13–62%)
School location: Rural (vs. non-rural)	56% rural (9 schools)

### Qualitative Analysis

Based on analysis, three main themes emerged: multi-level adaptability, student connection, and school contextual factors. These themes are defined below with the subthemes they capture. Supporting quotations from interviews for each subtheme are found in Table 3.

**Table 3:** Themes, subthemes, and definitions of qualitative analysis with supporting quotes from participant interviews

Theme	Theme definition	“6 C’s” coding family	Subtheme	Subtheme definition	Illustrative quote	Scope of theme
Multi-level Adaptability	A need to adapt from previous norms and standards to meet changing mental health needs across levels in the school setting	Cause, Consequence	Mental Health Shifts	Changes to mental health needs for students from pre-pandemic needs	“There were some students whose mental health needs really went up and those are the students who started having significant needs and didn’t have outside support, didn’t have great families, whose basic needs aren’t being met … and now all of a sudden mental health is, is one of those needs as well.”	Mental Health Services
			Implementation Changes	Changes to the implementation of specific mental health supports provided by SPs and schools	“I guess the biggest thing is that the co-facilitator I had leading that program was not able to come into the school building. So, he was basically pushing through onto Chromebook screens. And it was just, it was not very effective. It wasn’t a great experience.”	Mental Health Services, CBT Focused
			Hierarchy of Needs	Changes in the prioritization of the types or scope of support provided to or needed by students	“I personally went from dealing with the day-to-day crisis and working with students on how to, to deal with things that are outside of their control to calling students and parents trying to get them to get their homework done so that they didn’t fail their classes.”	Non-Mental Health Services/Mental Health Services
			Positive Byproducts	Unexpected, positive changes related to differences in student mental health need	“So, for some kids that was really a good feeling to not be in trouble all the time and not feel, you know, singled out one’s behavior plan. So, for some kids, I think it was really positive for their mental health.”	Mental Health Services
Student Connection	Ability to connect with students given changing student structure	Covariance, Condition	Accessibility	Establishing initial and continuing connection with students including, but not limited to, mental health support	“When we were [working from] home, I just couldn’t access and connect with students. They wouldn’t answer. I didn’t have the opportunity to pull ‘em outta class or drive to their house like I could previously.”	Non-Mental Health Services
			Student Engagement and Receptivity	Following initial connections between SP, promoting and maintaining student engagement in the delivery of mental health support	“That was the biggest challenge. I just remember it was really difficult to get students to engage.” “When we went remote, we would still set up the meetings [to talk about mental health], but then they wouldn’t show up.”	Non-Mental Health Services/Mental Health Services
			Communication	Modalities of contact to support changing delivery of mental health services, specifically	“And when you’re talking on the phone or through email, you don’t get body language, you don’t get to see how comfortable they feel with what you’re suggesting. And students are very good at hiding their feelings. And so, if you can’t have those face-to-face conversations, it became really difficult.”	Non-Mental Health Services/Mental Health Services
			Positive Byproducts	Unanticipated changes related to promising shifts in student connection and communication	“I had some students who weren’t really, even on my radar for needing mental health support who started coming to me.”	Mental Health Services
School Contextual Factors	Characteristics defining the unique contexts within which schools and SPs were operating	Context	School Setting & Culture	Factors related to the schools setting and culture related to supporting mental health delivery	“We ended up in a big disagreement with our principal about feeling very unsupported… you feel very undervalued all year and it’s hard to keep doing your job.”	Non-Mental Health Services
			School Professional Capacity	Ways in which the COVID-19 pandemic and related challenges impacted SPs and their ability to delivery support	“It was not a good year for providing those [mental health] services because it was hard for us as professionals too.” “I was so overworked. I would tell myself, ‘I just wanna hunker down, get through this day, and go home.’”	Mental Health Services
			Positive Byproducts	Helpful changes related to school-provided supports, processes, or structure	“I found teachers reaching out to me. In fact, I shared a couple of…pieces of information from the website with a few teachers 'cause they were concerned and like, 'okay, well how can I support this student?'”	Mental Health Services

### Multi-level Adaptability

The COVID-19 pandemic created a need for SPs to adapt from previous norms and standards to meet rapidly changing mental health needs and intervention capacity for student mental health in school settings. This adaptability was not just relevant to SPs’ own role but was seen across multiple levels including SPs interactions with students, parents, teachers, and administrators; adaptation to the mental health services, specifically CBT itself; and organizational priorities in times of rapid changes. For SPs who were not able to adapt to mental health delivery for a myriad of reasons (e.g., capacity, capability), it was difficult to provide CBT components or substantive mental health support. However, many SPs were able to adapt their approaches to mental health delivery to meet their students’ changing mental health needs. Sub-themes related to multi-level adaptability include mental health shifts, implementation changes, and hierarchy of needs. The subthemes characterized by this theme capture codes that fall under the cause and consequence categories of grounded theory.

#### Mental Health Shifts

COVID-19 led to major disruptions in students’ lives which both amplified mental health distress that students had been experiencing prior to the pandemic and brought up new needs in students. One SP said, “*Across the board*,* I’m gonna say there was a shift in their mental health needs because even kids that beforehand had high ASO [Assessment of Survivor Outcomes] scores or high anxiety*,* it got worse.*” SPs also noted that COVID-19 frequently led to co-occurring needs (i.e., anxiety, depression, behavioral problems, domestic violence, eating disorders, access to food and health services). On top of this shift in the underlying factors influencing increased student mental health need and the number of students that could benefit from mental health support, the change of structure to delivering support was made worse; this shift in day-to-day interactions and support capabilities contributed to worsen student mental health outcomes. When talking about the change in students’ mental health needs, a SP described, “*The access to support was more difficult and challenging in many ways*, *'cause even for those kids that were here*,* everybody’s masked up*,* the whole environment was different. Everybody was more isolated. There was way less dialogue*,* way less interactions*.” Responding to these shifts in mental health needs and focus required an adaptability from SPs that was not as salient prior to the pandemic.

#### Implementation Changes

Due to COVID-19, SPs were unable to implement group CBT sessions as they were trained. As a result, some SPs changed their implementation of CBT to respond to shifting needs and situations. One strategy that SPs used was modifying the modality of CBT delivery. While many SPs delivered CBT in a group setting before the pandemic, some SPs adapted materials to be used with students individually following COVID-19-related shutdowns. An SP described, “*I started doing a lot of one on one with students. Using TRAILS didn’t change*,* it just changed in the fact that it wasn’t as structured*.” A second strategy that SPs deployed was using only some CBT components and TRAILS materials that met their students’ needs, as opposed to full implementation of the TRAILS program. An SP reported, *“It was very hard to get students to engage fully. I probably used more self-care and COVID-19 related components that were provided by TRAILS versus any of the real meat of the curriculum with the more in-depth CBT strategies.*” While SPs felt the full program led to greater impact, pivoting to simplified versions of CBT or TRAILS-provided COVID-19-related materials allowed for delivery of some mental health support as opposed to none. Third, some SPs learned that engaging students in a virtual CBT group was difficult due to lack of engagement, technology issues, and changes in implementation support. Instead of continuing CBT groups virtually, some SPs instead waited until school was back in person and *“just picked back up and we started over with Module One.*”

#### Hierarchy of Needs

The type of intervention SPs provided changed in response to shifting needs. SPs told us that, especially at the start of the COVID-19 pandemic, they had to operate in ‘Survival Mode’. It was critical that they adapted their role to serve the immediate health, safety, and academic needs of their students. Once those immediate needs were being met, SPs could pivot to delivering CBT. One SP shared how she adapted to new student needs: “*I would set up Zooms and we would have conversations and we would work on plans*,* how to get through dealing with a parent who expects them to do all the chores*,* take care of their siblings*,* and do all of that and didn’t care that they still had to do homework.*” SPs also shared that, in addition to increases in students’ depression and anxiety increase, other concerns like domestic violence and eating disorders also arose. SPs were required to navigate and address multiple concerns in unfamiliar contexts, all while concurrently responding to evolving student mental health needs.

#### Positive Byproducts

While almost all SPs interviewed discussed the extreme detriment COVID-19 had on student mental health, some participants discussed unexpected changes they saw regarding their students’ mental health during COVID-19. Specifically for students with anxiety about physically being at school, social anxiety, or distress around coursework and grades, some SPs reported improvements in these stressors when schools were shut down due to COVID-19 or offering remote learning opportunities. Regarding a few of her students, an SP shared,

Everybody was at home last year. So, it was like we had no behavior problems. We had no kids getting kicked outta class, suspended, et cetera. So, for some kids that was really a good feeling to not be in trouble all the time and not feel, you know, singled out one’s behavior plan. So, for some kids, I think it was really positive for their mental health.

Another positive, unanticipated outcome was related to the overall change in environment, and its implications for student mental health. By transitioning to a virtual space, some school-based contextual factors were no longer perceived as problematic mental health concerns for students. Students who experienced school-based bullying, negative peer interactions, and unsupportive school climate felt that the virtual transition was better for their overall mental health. One SP described the feedback from some students regarding the virtual environment and their mental health:

The feedback I got from kids who I reached out [to] during that time was basically this break from school has been good for my mental health because I’ve escaped the drama, I was wrapped in by being around these codependent or toxic peers. So, there were actually…some positives for mental health coming out of that first shutdown.

In summary, the COVID-19 pandemic required SPs to exercise multi-level adaptability, transforming their approaches to meet evolving student mental health needs. While challenges were pervasive, particularly in maintaining engagement with students and delivering group-based CBT, some SPs successfully adapted their methods, prioritizing immediate student needs and turning to individual CBT delivery. The shift to remote learning environments also yielded unexpected mental health benefits for certain students, reducing sources of anxiety and negative interactions present in previous in-school settings. These findings highlight both the complexities and the potential for some positive transformation within school-based mental health services during times of crisis.

### Student Connection

One area that SPs continued to navigate during the COVID-19 pandemic was the ability to connect with students. Transitioning away from in-person school settings to virtual school presented some unforeseen challenges with respect to how to maintain student contact. In particular, SPs described how they navigated student accessibility, engagement, and communication. These three areas introduced challenges and opportunities to find ways to continue to reach students, despite the unique circumstances. In some instances, SPs described creative solutions for mitigating these challenges. However, other SPs reported experiencing barriers that prevented them from supporting students during the virtual shift. Below, the subthemes characterized by this theme capture codes that fall under the covariance and condition categories of grounded theory.

#### Accessibility

SPs developed creative and swift approaches to accessing students, and in some cases, experienced substantial hurdles in establishing lines of communication. Some SPs reported that student access was further limited by geographical circumstances, challenging home environments, and technological restrictions. One SP described obstacles in establishing an initial connection with their students post-shutdown:

So, we did our best to be connected to students during the virtual time, but that was hard. Some of our students didn’t have access to great internet. They didn’t have supports at home. They didn’t have the schedule. So…I was trying to contact students through email over the phone. So… that was challenging.

Other SPs, however, explained how the virtual situation positively impacted accessibility:

It’s much easier to [regularly reach students virtually]. And we’re… there for crisis situations as well, but not necessarily even crisis, but [students are] more just like, ‘Oh, I need to seek out mental health supports right now. Like I need to talk to someone about what’s going on’ and we might get a message and I might be able to respond pretty quick.

#### Student Engagement and Receptivity

SPs described challenges encountered in both establishing initial contact for mental health delivery and also maintaining engagement after contact was established. For example, one SP noted difficulties with overall student engagement: *“That was the biggest challenge. I just remember it was really difficult to get students to engage.”* Another SP described how students would fail to engage in any capacity, and how this was perpetuated by interactions in a virtual capacity:

So just trying to kind of get them on the screen and engaged because this is way better than what I was doing face-to-face with Plexiglas and masks. So, when I could get kids on the screen doing Google Meets it was really good. The issue was that during their virtual time they didn’t have to engage with anyone. There was no teacher checking up on them and their online facilitator would just send them an email and they would write back and say, “I’m good” or “I’m working”. And so, I think they felt isolated and this was a lot for them. So, the kids that I could get on the screen, it was great. And we would talk about things…but it was far fewer than I would’ve liked.

Successfully establishing and maintaining engagement was a challenge navigated by SPs, and required adaptation, creativity, and persistence and was described as a crucial aspect to overcome to support the unique mental health needs of students.

#### Communication

A final subtheme expressed by SPs regarding student connection was communication. Specifically, many SPs identified the shift from in person to other modalities as a particular challenge in reaching and fostering connection with students. In particular, several SPs expressed belief that technology-driven means of communication created inauthentic experiences that allowed students to mask their true feelings, and also limited information conveyed through non-verbal communication or body language. This prevented SPs from understanding how best to comprehensively support students. One SP commented on the importance of body language as part of holistic communication:

“And when you’re talking on the phone or through email, you don’t get body language, you don’t get to see how comfortable they feel with what you’re suggesting. And students are very good at hiding their feelings. And so, if you can’t have those face-to-face conversations, it became really difficult.”

The infrequency and inconsistency of communication was also described as a pain point by SPs. Without the regularity of face-to-face contact within the school, it was more difficult to engage in frequent and regular conversations or interactions with students, which made it challenging to identify student mental health needs, or how to best connect and communicate with their students.

#### Positive Byproducts

Despite the challenges presented as a result of the shift to online mental health delivery, some SPs described unexpected affordances of the virtual transition for connecting with students. For example, changing the delivery context allowed some students to become more aware about the availability of mental health resources, or aware of their own mental health needs and seek help. For example, one SP commented, *“I had some students who weren’t really even on my radar for needing mental health support who started coming to me*.” Another SP noted that some students indicated that they had not previously sought out mental health support because they did not know where the counseling office was located, or what services the office provided.

SPs also shared how more teachers were seeking collaborative opportunities to assist with student mental health. SPs described how more teachers were seeking resources from them in the virtual space as compared with their school-based experiences. With an increase in students seeking help, more teachers became aware of the growing student need and wanted to equip themselves with sufficient resources: *“I found teachers reaching out to me. In fact*,* I shared a couple of…pieces of information from the TRAILS website with a few teachers 'cause they were concerned and like*,* okay*,* well how can I support this student*?” With the increase in communication and collaboration between teachers and SPs regarding mental health resources, there were emerging opportunities to support student needs and create more positive ways to connect with students overall.

In summary, SPs faced significant challenges in maintaining student connection during the COVID-19 pandemic as they transitioned to virtual environments. Despite barriers, SPs employed creative strategies to reach students. These findings emphasize both the obstacles and the potential for enhanced outreach in a remote setting, underscoring the importance of adaptability and innovation in supporting student mental health.

### School Contextual Factors

Contextual factors related to schools, the school community, and those delivering mental health services influenced the success of mental health care delivery in school environments changed by COVID-19. The subthemes characterized by this theme, including school setting and culture and SP capacity, capture codes that fall under the context category of grounded theory.

#### School Setting and Culture

Multiple factors defined the unique setting in which schools were operating during COVID-19. These include the schools’ urban/rural status, resource availability, funding, administration support, overall school and community culture towards mental health, and others. In turn, these factors impacted schools’ ability to respond to changes caused by COVID-19 regarding the delivery of mental health services and overall support to students. Administration support was a commonly cited factor that allowed SPs to continue delivering CBT following COVID-19 or to engage with any type of support more broadly. An SP told us that “*one thing that is a benefit is our building administration. Anything we want to do or try*,* they’re usually very supportive of that*” showing that administrative support was key for trying new approaches or maintaining other forms of support to students. When discussing why another school was unable to deliver TRAILS materials, an SP disclosed “*our demographic in our school district is pretty*,* uh*,* there’s a lot of challenges. We are very high poverty*,* very rural*,* have limited resources. So*,* there’s always been that challenge there.*” This SP went on to share that “*a challenge of parent involvement has always been a struggle in our school and has just been made so much harder since the pandemic.*” These findings emphasize both the obstacles and the potential for SP outreach that vary across settings and highlight how a supportive school culture and setting can significantly bolster the ability of SPs to navigate challenges and maintain their commitment to student mental health.

#### School Professional Capacity

One important theme that repeatedly emerged in conversations with SPs was regarding their own capacity to engage and their personal impacts of COVID-19. Many SPs have roles that involve more than delivering mental health services to students. For example, in addition to providing counseling and mental health support one SP is “*also the district’s Title VI coordinator. Basically*,* my whole job in the district is to just keep tabs on the social*,* emotional health and wellbeing of our kids*.” However, during the pandemic, SPs were often asked to step into even more roles that schools needed, almost always at a detriment to time dedicated towards providing direct mental health support to students. These additional tasks were one of many factors contributing to SP burnout at this time. One SP shared:

I wish that there were two of me < laugh>, I wear a lot of hats. So, for instance, they have me also covering not only the mental health portion, but also the kids who are failing academically. Since COVID, they want me doing academic support, which isn’t really my wheelhouse, but, you know, I try as best as I can to fit in where I can. But it stretches me thin. I really wanted to try to do [TRAILS] groups by the end of the year and it just didn’t pan out because I just didn’t have the time for it. So, I don’t even know what would fix that other than some like time management or, um, more resources to cover more areas so that I could focus and do one area very well.

Many SPs reported experiencing burnout, though its manifestations varied both across individuals and at different stages of the pandemic. Some SPs talked about feelings of stress caused by feeling they were unable to do their job well. Others felt there was just too much being asked of them. When talking about why it was difficult to deliver CBT, an SP shared, “*in addition to the normal barriers [to TRAILS delivery]*,* it also takes professional energy*,* emotional energy*,* time and investment.*” Throughout this many SPs shared that they felt their efforts were undervalued and they were left feeling unsupported through one of the most challenging parts of their careers. An SP shared a story relating to this point:

We ended up in a big disagreement with our principal about feeling very unsupported and the principal was quite upset that that’s how we felt. And we were upset and the year ended badly because there were just so many points during the year that we were like, ‘Nobody notices this. Nobody notices that. Nobody [notices] we are working so hard.’ You feel very undervalued all year. Even though we were allowed to do whatever we wanted, it didn’t mean that we were feeling maybe good or successful or on top of things.

Throughout this, many SPs mentioned how hard it was dealing with stress and anxiety related to COVID-19 themselves. An SP summarized, “*it was not a good year for providing those services because it was hard for us as professionals too*.”

In summary, the success of mental health service delivery in the context of COVID-19 was influenced by multiple, school-specific factors. These elements determined the extent to which SPs could implement necessary modifications and maintain student support. Additionally, the personal and professional capacity of school professionals was significantly tested, as they were stretched thin by multiple responsibilities and the emotional toll of the pandemic. Despite the supportive environments in some schools, many professionals faced burnout and feelings of undervaluation.

## Discussion

In this study, we used interview data from 19 SPs representing 16 schools that were involved in an implementation trial and receiving support to implement an evidence-based mental health practice, CBT, in their schools to highlight the critical role of SPs in maintaining mental health support to students during times of crisis. Our goal was to better understand how SPs navigated evidence-based mental health service delivery during the unprecedented challenges of the COVID-19 pandemic. Through qualitative analysis of these interviews, we identified three key themes shaping SP experiences: multi-level adaptability in response to changing student and school needs; the ongoing challenge of maintaining meaningful student connection; and the influence of varying school contextual factors. These themes highlight both the complexity and nuance of modifying and sustaining mental health supports in times of crisis, and underscore the importance of flexibility, creativity, and a supportive environment for effective service delivery in challenging circumstances.

A pattern that emerged throughout our findings was the importance of SP adaptability and agile responsiveness. SPs not only had to reach and engage students under rapidly changing circumstances, but also needed to continually implement ongoing, multi-level modifications to sustain delivery of mental health programming with students. Examples of modifications to pre-pandemic practices included trying new approaches to maintain connections to students despite physical school closures, modifying specific CBT components to meet new student needs brought about by the pandemic and resultant changes, and cultivating collaborations with teachers and administrators to work around new barriers. SPs reported that these efforts resulted in more effective student engagement and provision of support. They also often demonstrated innovation and tenacity in reaching students, particularly those with urgent mental health concerns.

SPs developed modified approaches out of necessity, but implications highlight the potential value of institutional adaptability, even under stable conditions. This aligns with established literature on organizational readiness, which presents how an organization’s capacity can dictate how they absorb and respond to change, and the dynamic sustainability framework, which emphasizes ongoing adaptation to improve sustained intervention use (Chambers et al., 2013; Weiner, 2009). SPs learned that certain mental health delivery practice changes were more successful than others. While COVID-19 created a range of crises that required a repertoire of modified response strategies, lessons learned from this study could extend to additional contexts including virtual learning, teletherapy, and other emerging changes to in-person mental health delivery. Fostering an environment where flexible responses are encouraged and supported may strengthen both day-to-day practice and crisis preparedness.

The largely individual and ad hoc nature of SP changes in practice underscores the need for more systematic and planned methods to capture continued modifications and measure effects on student mental health. Research efforts to methodologically capture the content and process modifications SPs make could detail the rationale, timing, and decision-making processes underlying identified changes and encourage future planned adaptation. Specifically, the Framework for Reporting Adaptations and Modifications (FRAME) and the Framework for Reporting Adaptations and Modifications to Evidence-based Implementation Strategies (FRAME-IS) systematically capture adaptations and modifications made to either evidence-based practices or implementation strategies, respectively (Miller et al., 2021; Wiltsey Stirman et al., 2019). FRAME provides a tool for understanding and detailing modifications made directly to evidence-based practices, such as CBT adjustments SPs made for remote delivery. In contrast, FRAME-IS focuses on capturing changes to implementation strategies, for example, adjustments to TRAILS-provided implementation support during disruption. These frameworks can illustrate not only what was changed, but also why, when, and under what circumstances, allowing for deeper understanding and replication in other future situations. Addressing this complexity could enhance clarity of causal linkages and deepen understanding of how intervention and implementation strategies specifically contributed to mental health delivery outcomes amidst rapidly changing contexts in schools.

Finally, our findings reinforce the need to understand school-specific context to support adaptation and sustain the work of mental health SPs. Supporting SPs in their roles during times of rapid change will ultimately necessitate the development of strategic implementation frameworks that incorporate flexibility and cooperation across the school community. Future research efforts could examine the nuance of the perceived barriers experienced by SPs and develop evidence-based recommendations for addressing and overcoming these barriers. As schools consider lessons learned from the COVID-19 pandemic, these insights lay the groundwork for creating more resilient and responsive mental health support systems.

This research can clarify effectiveness of alternative or adaptive delivery methods of school-based mental health support and identify areas for improvement. While the immediate relevance of this study concerns preparing for future school disruptions to standard operations, its broader significance extends to other contexts. As educational environments increasingly integrate technological advancements, understanding adaptive strategies becomes essential for effectively deploying strategies like distance learning and telehealth initiatives (Goddard et al., 2021; Nuryana et al., 2023). As mental health awareness grows and more students receive mental health services in school settings, the utility of these findings for informing future approaches is increasingly crucial and applicable. Developing a comprehensive understanding of how to support students, even during large-scale disruptions, is imperative for ensuring service delivery can be successful despite challenges that inevitably can affect student success and well-being.

### Implications

This study contributes to understanding challenges involved in reaching and engaging students to support their mental health needs in response to significant disruption to school structures and practices. While broadening research and practitioner understanding of the difficulties of supporting students experiencing ongoing trauma, the experiences shared through this study illustrate a range of modifications SPs made. This research highlights the often-overlooked dedication and compassion of SPs who are committed to delivering and sustaining student mental health support, even in times of global crisis. Moreover, this work presents a historical record that captures efforts and inventive solutions employed during a tumultuous time. This research is unique in specifically highlighting the extraordinary measures SPs engaged in a trial to implement CBT employed to ensure continued mental health support for students.

This study also describes and identifies perceived barriers to mental health care facing students who were experiencing trauma, underscoring the necessity of building a more proactive mental health support system within educational settings with capacity to engage students across a variety of contexts. Insights offer guidance for adapting to future advancements or shifts in mental health delivery, providing groundwork that can help schools become more agile and prepared to meet the evolving mental health needs of their students.

Finally, this research serves as an important reflective opportunity for creative and innovative approaches to school-based research. By leveraging the voices and experiences of those most involved in supporting student mental health in an unusual and rapidly changing environment, these findings provide a critical window into how service delivery in school contexts functions. This carries implications for future delivery both in times of crisis, and potentially to better support students overall. Findings suggest that crisis situations or evolving mental health delivery approaches require imaginative solutions that can potentially pave the way for changes in accessibility and integration of mental health services within educational settings overall.

### Limitations

The representativeness of this study has limitations. This study was carried out in a limited number of Michigan high schools. While there was a statewide physical school shutdown, each school district in Michigan responded differently to COVID-19, and opinions expressed in these interviews may not have fully captured the range of experiences of or changes to mental health service delivery. Furthermore, only SPs enrolled in the ASIC study that were successfully delivering CBT to their students prior to COVID-19 were eligible for participation. SPs who were not successfully delivering CBT or who did not participate in the ASIC study may have different experiences. Additionally, participating SPs opted into this interview study; those who did not participate may have experienced different or even greater barriers that may have implications for both their ability to deliver mental health treatment and their capacity for adaptability. This study also only captured the perspectives and actions of SPs at one specific point during the pandemic and their implementation may have shifted over time. Finally, as this study was designed to capture SPs’ self-reported experiences and perspectives, the level of detail regarding specific CBT delivery adaptations was limited to what participants chose to share during interviews. As a result, our findings reflect the broad strategies and overall approaches described by SPs, rather than detailed procedural modifications.

## Future Directions

Understanding how schools continue to deliver CBT and other mental health services in the aftermath of COVID-19 related disruptions can provide insights into the resilience and effectiveness of these interventions, and overall school operations. As schools expand delivery offerings to meet the evolving needs of learners, learnings from this research can help inform the delivery of virtual learning, teletherapy, and other deviations from in-person mental health delivery. Future research efforts could examine the aspects that contribute to the sustained use of CBT, including ongoing training and support for SPs, organizational factors, and resource allocation.

While this study focused on the perspectives of SPs, it is also critical to assess the effect of modified mental health service delivery on student outcomes, ideally on both academic and mental health outcomes. By evaluating the effectiveness of the modified interventions, researchers can identify adaptation strategies and mechanisms that have the greatest positive outcomes for student well-being and academic success. These avenues of inquiry can further advance the understanding of effective and adaptable approaches to supporting the mental health needs of students during times of crisis.

This research initially focused on how CBT delivery in schools changed during COVID-19. However, broader conversations regarding mental health emerged during interviews with SPs. Enhancing school-based mental health supports can benefit from a more comprehensive implementation approach, considering how various facets of implementation strategies affect mental health outcomes in school settings. The shift from modification of existing practices to planned adaptation highlights the importance of tailoring interventions to diverse contexts and needs. By translating adaptive strategies from other spaces (i.e., clinical settings) to the school setting, researchers and practitioners can enhance schools’ ability to address student mental health challenges more holistically. This broader perspective can not only improve effectiveness of mental health interventions but also ensure that future approaches are more inclusive and responsive to the complex realities faced by students and educators.

## Conclusion

These findings have implications for the development of best practices and policies in delivering mental health services during future public health crises or exogenous shocks to the school system. However, before changes to mental health services in these situations can reach students, strategies to better support access to students, student engagement, and communication need to be developed and prioritized. Successful mental health interventions in these contexts depend on a thorough understanding of the unique school environment and the capacity to support the continued work of mental health professionals. This research offers a foundational framework for adapting implementation of evidence-based practices in school-based mental health care during times of crisis. Future efforts should focus on operationalizing these insights to build resilient systems that can proactively respond to the dynamic and evolving needs of students, schools, and communities in crisis situations and beyond.

## Supplementary Information

Below is the link to the electronic supplementary material.

Supplementary Material 1
