# Supplementary material for: Meeting Student Mental Health Needs in Survival Mode: A Qualitative Analysis of School Professional Mental Health Delivery at Michigan High Schools in Response to COVID-19
Source: School Ment Health. 2026 Jan 13;18(1):243–58. doi: 10.1007/s12310-025-09838-y (PMC12971736; doi:10.1007/s12310-025-09838-y)
Supplement: Supplementary file 1 — Supplementary Material 1 [file 12310_2025_9838_MOESM1_ESM.docx]

**Supplemental Table 1. Semi-Structured Interview Guide**

**Introduction Questions**:

- Can you tell me about your role in your school?
- *[If applicable]* Has your role formally changed since our last conversation?
  - *[If yes]* Can you tell me what your new role entails & when this change happened?
- Can you tell us briefly how your school has handled instruction since the initial March 2020 statewide school shutdown? *For the purposes of this interview, “this school year” will refer to March 2020-June 2021*. Have you/your school resumed in-person instruction &, if so, when did that occur?
  - During the period your school was entirely virtual, how did your interactions with students change? How did you manage these interactions?
  - *[If hybrid education is still ongoing]* What steps, if any, have you taken to help manage interactions with your students that are still engaging entirely or primarily virtually? Are there steps you take to engage with these students that are different from students that you see in person?

**Question 1.** Generally speaking, how did COVID-19 impact your work during the 2020-2021 school year?

- What were the biggest challenges you faced in your role in response to COVID-19? How did these challenges compare to those you faced prior to COVID-19?
- What steps were you able to take to address these challenges? Were there challenges you were not able to overcome over the year?
  - What support or additional resources, if any, did you receive to help you address these new challenges?
  - What support would you have liked to receive? What impact do you think these supports could have had on you or your students?

**Question 2.** Now I would like to speak specifically about your delivery of mental health care to your students. As a participant in the ASIC program, we know that you were engaged with the resources provided by the TRAILS program and also that prior to the March 2020 school shutdown you were delivering CBT to your students fairly regularly. Can you tell us a little about how your delivery of CBT and other mental health care to your students changed and/or what new challenges you experienced in delivering mental health care to your students:

- First, immediately following the statewide school shutdown in March 2020 (i.e., remainder of the Spring 2020 semester)?
- And second, for the 2020/2021 school year?
- What factors most impacted changes to your delivery during these two time periods?
- What steps, if any, were you able to take to overcome or address these new challenges?
- What challenges or barriers were you not able to address? What impact do you think these new challenges or barriers had on your ability to do your job? What about their impact on your students’ mental health, well-being or academic outcomes?
- With respect to your students, what changes did you see, if any, in their needs for mental health care or support, both immediately after shutdown and during the 2020/2021 school year?
- What resources or supports, if any, were helpful to you in managing either these new challenges or your students’ changing mental health needs?
  - Did you make use of any of the resources provided by the TRAILS team? *[If yes]* Which did you find most useful?
- What resources or supports would you have liked to receive? What impact would these resources or supports have had on you or your students’?

**Question 3** Now thinking specifically of the CBT delivery that you were reporting under the ASIC trial:

- Can you tell me briefly about your CBT delivery during the winter/spring prior to the March 2020 school closures? How were you delivering CBT, how often, to how many students, etc?
- Were you able to continue to deliver CBT to your students:
  - During the Spring 2020 semester? Can you tell me briefly about this delivery?
  - During the 2020-21 Academic Year? Can you tell me briefly about this delivery?
    - *[If yes]* Can you tell me a little about any changes you had to make to your delivery? (e.g., virtual delivery, fewer group sessions, etc)
    - *[If yes]* What helped you or encouraged you to continue this delivery? What worked well? Alternatively, did you try anything that did not work?
    - [*If yes*] What components of CBT did you find particularly useful when delivering mental health care to your students during this time?
    - *[If yes]* In your opinion, how effective was CBT for your students? Could you share what impact, if any, these changes had on the effectiveness of the intervention? Did these changes impact your comfort in delivering the intervention to your students?
    - *[If no]* Can you tell me a little about the factors that kept you from being able to continue delivering CBT? What type of resources, if any, might have helped you to deliver CBT under these circumstances?
    - Hypothetically speaking, if COVID-19 didn’t occur, were you planning on continuing your use of TRAILS?
    - Did you make use of any of the TRAILS resources when considering whether or how to continue delivering CBT?
      - *[If yes]* Were there specific resources that you found especially helpful? Were there resources that you would have liked TRAILS to provide?

**Question 3.1** For SPs that specifically delivered CBT virtually:

- - *[If SP was able to deliver CBT Virtually]* What strategies did you use to deliver CBT virtually to your students? What strategies worked well? What aspects of BT were more difficult to deliver? How was the quality of your delivery impacted?
  - What, if anything, made it easier to deliver CBT via virtual learning environments?
    - Did you use any TRAILS provided resources in your virtual learning environments?
    - What other resources or supports could have impacted your delivery?
  - *[If SP was NOT able to deliver CBT Virtually]* What prevented you from delivering CBT virtually to your students? What might have helped you in your delivery?

**Question 4.** Other than CBT, did you use any other mental health therapies or treatments with your students during the 2020-2021 Academic Year? Can you tell us about those?

- Were these therapies or treatments that you had used before?
- Were there some therapies or treatments that worked better with the circumstances--e.g., with the virtual school environment? To accommodate changing or new student needs?
- Were there any that were made more difficult or seemed less useful given changing circumstances?

**Question 5.** Thinking ahead to the 2021-2022 academic year:

- To the best of your knowledge, what do you expect instruction to look like? E.g., nearly all in-person, nearly all virtual, hybrid?
- What will the next school year look like in terms of your mental health delivery?
  - Are you planning to continue or restart delivering CBT or other TRAILS materials to your students? What are you hoping this will look like?
  - What, if any, changes are you anticipating with respect to changes in student mental health needs? Have you considered how you might accommodate these changing needs?
- What supports would be most useful for you in preparing for mental health delivery next fall?

**Wrap-up Question.** Thank you for your time. Before we conclude, is there anything else you would like to share that we have not covered in this interview?
